# Supplementary figures and images for: Behavioral phenotyping of mice lacking the deubiquitinase USP2
Source: PLoS One. 2021 Feb 23;16(2):e0241403. doi: 10.1371/journal.pone.0241403 (PMC7901773; doi:10.1371/journal.pone.0241403)

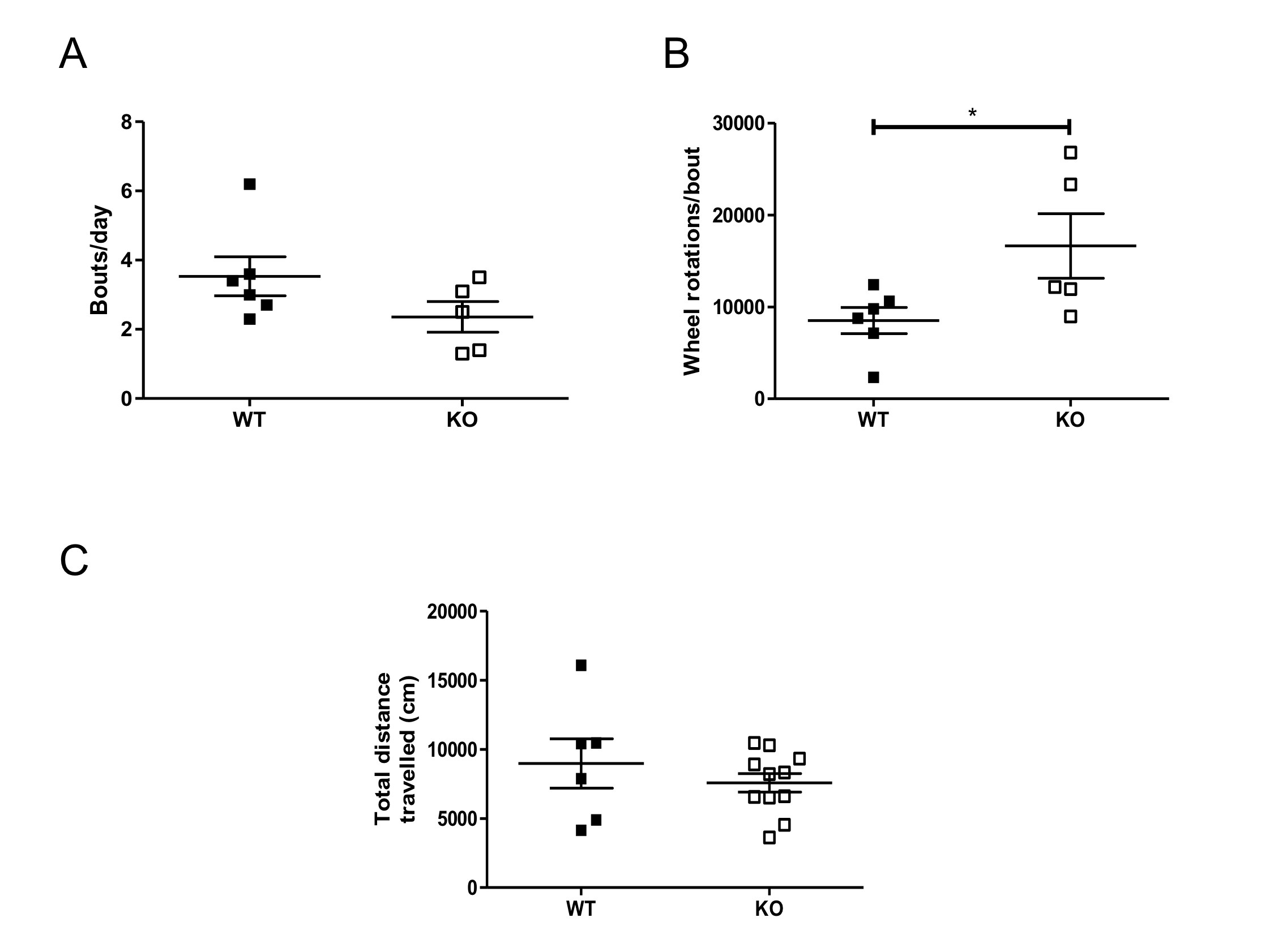

Supplement: S1 Fig — (A, B) Quantification of averaged wheel running activity over 10 days: number of bouts of activity per day (A) and number of wheel rotations per activity bout (B). (C) Open field measurement of total distance traveled by WT and Usp2 KO mice as a measure of general locomotion. Individual data points represent independent mice (Wheel-running activity, n: WT = 6, KO = 5; Actimetry, n: WT = 6, KO = 11) and data are represented as mean ± SEM. Unpaired two-tailed t-tests, * p < 0.05. (TIF) [file pone.0241403.s001.tif]

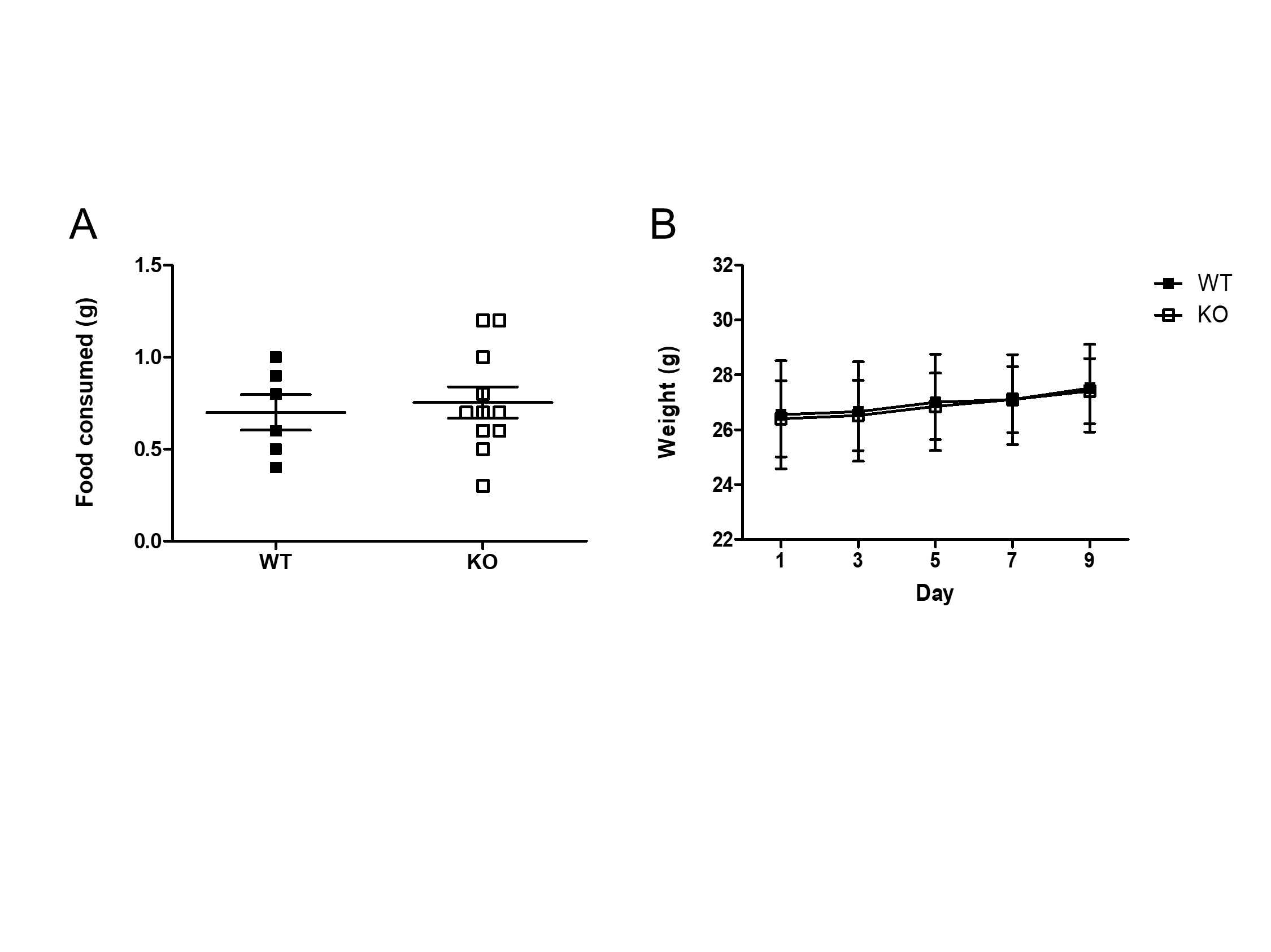

Supplement: S2 Fig — (A) Quantity of food consumed within 10 minutes post-NSF test, in the home cage. (B) Weight of the mice over 9 days following the NSF test. Individual data points represent independent mice (n: WT = 6, KO = 11) and data are represented as mean ± SEM. Unpaired two-tailed t-tests (A) or two-way ANOVA (B), all n.s. (TIF) [file pone.0241403.s002.tif]
